# The effect of corticotomy on the compensatory remodeling of alveolar bone during orthodontic treatment

**DOI:** 10.1186/s12903-021-01492-5

**Published:** 2021-03-19

**Authors:** Yi-Fei Wu, Hong-Ming Guo

**Affiliations:** Department of Orthodontics, Capital Medical University, School of Stomatology, Beijing, 100050 China

**Keywords:** Corticotomy, Compensatory remodeling, TGF-β1

## Abstract

**Background:**

This study aimed to explore whether compensatory remodeling of the alveolar bone surface occurred during the buccal palatal movement of orthodontic teeth. We preliminarily explored whether corticotomy could activate or accelerate osteogenesis in the alveolar bone surface by measuring the expression of TGF-β1 (transforming growth factor-β1), which can facilitate the proliferation and differentiation of osteoblasts and regulate the maturity and formation of bone.

**Methods:**

Sixty 10-week-old male Wistar rats were selected. In the orthodontic group, 20 rats were implanted with a constriction device between the maxillary first molars under general anesthesia. In the corticotomy group, 20 rats were implanted with a constriction device, and a palatal incision was made to penetrate the cortical bone. In the control group, 20 rats underwent no experimental operation except general anesthesia. After 1, 3, 5 and 7 days, the maxillary first molars and the surrounding alveolar bone were harvested, and coronal sections containing the apical mesial buccal root were prepared and observed using tetracycline fluorescence, HE staining and immunohistochemical staining for TGF-β1. Image-Pro Plus software was used to assess the immunohistochemical results, and SPSS 22.0 statistical software was used to analyze variance and perform the LSD test.

**Results:**

The tetracycline fluorescence results showed that in the periosteum near the apical region, an obvious fluorescence signal was observed in the orthodontic group and the corticotomy group compared with the control group. In the orthodontic group and corticotomy group, HE staining showed that the morphology was similar to cube-shaped. The immunohistochemical results showed that TGF-β1 was significantly increased in the periosteum near the apical region in the orthodontic group and corticotomy group, and there were significant differences among the three groups. In addition, the expression of TGF-β1 in the periosteum in the orthodontic group and the corticotomy group gradually increased over time, reaching a peak on day 5 and slightly decreasing on day 7.

**Conclusion:**

Osteogenesis occurred on the alveolar bone surface during the buccal palatal movement of orthodontic teeth, and corticotomy had a positive effect, and TGF-β1 was involved in this process.

## Background

With the clinical application of CBCT, an increasing number of orthodontists have begun to pay attention to fenestration and dehiscence before and after orthodontic treatments. According to traditional orthodontic biology, when teeth move toward the lip and tongue, alveolar bone absorption and accretion occur on the pressure and tension sides. In addition, compensatory accretion and absorption occur on the surface of the corresponding alveolar bone to maintain constant alveolar bone thickness. However, a large number of CBCT measurements have shown that the alveolar bone does not move equally with the teeth following the removal of first premolars with retraction of the anterior teeth [1]. The palatal alveolar bone thickness is generally decreased, while the labial alveolar bone thickness remains unchanged or exhibits only a small amount of accretion. Generally, the alveolar bone is mainly absorbed, and accretion is very limited [2, 3].

Corticotomy was introduced by Kole in 1959 as a technique to accelerate tooth movement by injuring alveolar bone [4]. Cortical osteotomy or perforation with or without flaps is performed around the teeth that need to be moved, effectively shortening the time of orthodontic processes, such as the retraction of anterior teeth [5–7], intrusion of overerupted molars [8, 9], expansion of the dental arch [10, 11], treatment of open bites [12, 13], traction of impacted teeth [14, 15] or moderation of dental crowding [16, 17]. Currently, the mechanism of tooth acceleration is believed to occur via the regional acceleratory phenomenon (RAP) [18]; that is, after cortical bone is damaged, the metabolism of local soft and hard tissues is accelerated, the alveolar bone around the tooth initiates rapid demineralization and remineralization processes, and the tooth can rapidly move under the action of orthodontic force. Studies have shown that after corticotomy, the hyalinization area on the pressure side of the periodontium was decreased, direct bone resorption occurred on the surface of the lamina dura in the early stage, and osteoblast accumulation and active osteogenesis occurred on the surface of the tension side [19–22]. However, the role of corticotomy in the compensatory remodeling of the alveolar bone surface has not been widely studied.

Oral and maxillofacial bone growth mainly relies on periosteal ossification, blood vessel formation in the bone area, mesenchymal cell proliferation and accumulation, membrane formation, and the differentiation of mesenchymal cells into bone cells; in addition, osteoblasts secrete osteoids to encase themselves and form bone cells, which in turn leads to early bone calcification [23]. In the late periosteum, new cells are constantly formed, and extracellular matrix is secreted and gradually mineralized into hard tissues. Studies have shown that in response to mechanical stimulation, osteoblast proliferation is enhanced, and osteoblasts can produce active transforming growth factor-β1 (TGF-β1) [24]. Activated TGF-β1 also activates latent TGF-β1 complexes. TGF-β1 stimulates mesenchymal cell accumulation and proliferation, facilitates vascular proliferation, regulates cell proliferation and differentiation, and plays a role in the induction of new bone formation by bone morphogenetic protein (BMP) [25]. Therefore, to a certain extent, TGF-β1 expression can be considered a reflection of local bone metabolism and bone remodeling.

In this study, a rat model of buccal palatal movement toward teeth was established, and the changes in TGF-β1 expression in the periosteum of the alveolar bone surface near the apex were observed by histological analyses. We focused on the outer surface of the alveolar bone area to examine whether this area underwent compensatory accretion and absorption, as described by traditional orthodontic biology. Whether corticotomy can accelerate this process, especially osteogenesis, and thus slow or stop fenestration and dehiscence remains unclear.

## Methods

### Experimental animals and groups

All animals were provided by SPF Biotechnology Co., Ltd. (Beijing) and fed in the animal house of Beijing Stomatological Hospital.

Sixty healthy 10-week-old male Wistar rats, which had neither periodontal disease nor dental disease, were selected and randomly divided into three groups via a random number table (20 rats per group): the control group (group A), the orthodontic group (group B), and the corticotomy group (orthodontic constriction plus corticotomy) (group C). According to the different loading times, the rats were subdivided into a 1-day group, a 3-day group, a 5-day group and a 7-day group. Five rats were included in each subgroup. Note that confounding factors in the experiment were not expressly controlled.

### Methods

#### Establishment of a rat model of buccal and palatal tooth movement

**Fig. 1:**
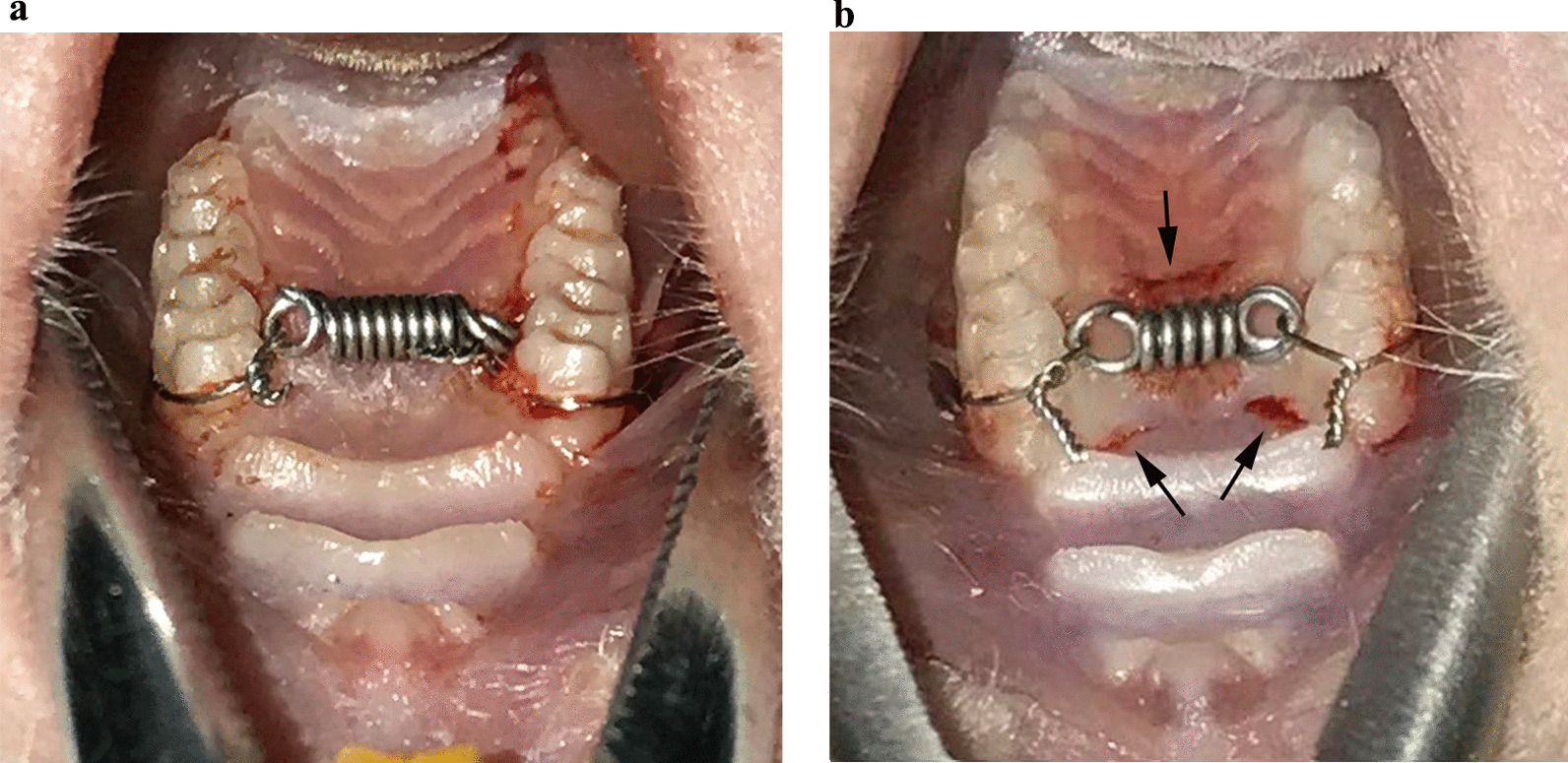
Palatal reinforcement device for rat teeth. (Figures were edited using Adobe Photoshop CS6, version 13.0*64.). **a** Intraoral view of rats in group B. **b** Intraoral view of rats in group C. The black arrows indicate three wounds cut with sharp surgical blades through the entire gingiva and cortical bone

After 1 week of adaptive feeding, rats in group B and group C were subjected to general anesthesia, and a palatal reinforcement device was attached to the maxillary molars. Nickel-titanium tension springs were ligated between the first maxillary molars on both sides, and a small amount of deformation was performed to generate a pull force to move the crown palatally. A wedge-shaped defect was generated by using a high-speed turbine to fix the ligation wire in the neck of the proximal tooth. A Vernier caliper and force meter were used externally to measure the elongation and force of the tension spring so that the first maxillary molars on both sides were subjected to a force of approximately 30 g directed to the palatal side (Fig. 1a). In group A, the rats were only subjected to anesthesia, but no orthodontic constriction was performed.

#### Establishment of a corticotomy model

In group C rats, the first maxillary molars on both sides were cut with a sharp scalpel through the entire gingival layer and the cortical bone after ligation of the force device in the mouth. Two wounds of approximately 2 mm in length and 2 mm in depth were made in the mesial surface, and one wound of approximately 3 mm in length and 2 mm in depth was made in the distal surface (Fig. 1b).

All the rats were fed soft food so that the wounds and devices in their mouths did not affect food intake. These devices were evaluated every day. Rats whose devices were not in the correct position were excluded.

#### Specimen collection

**Fig. 2:**
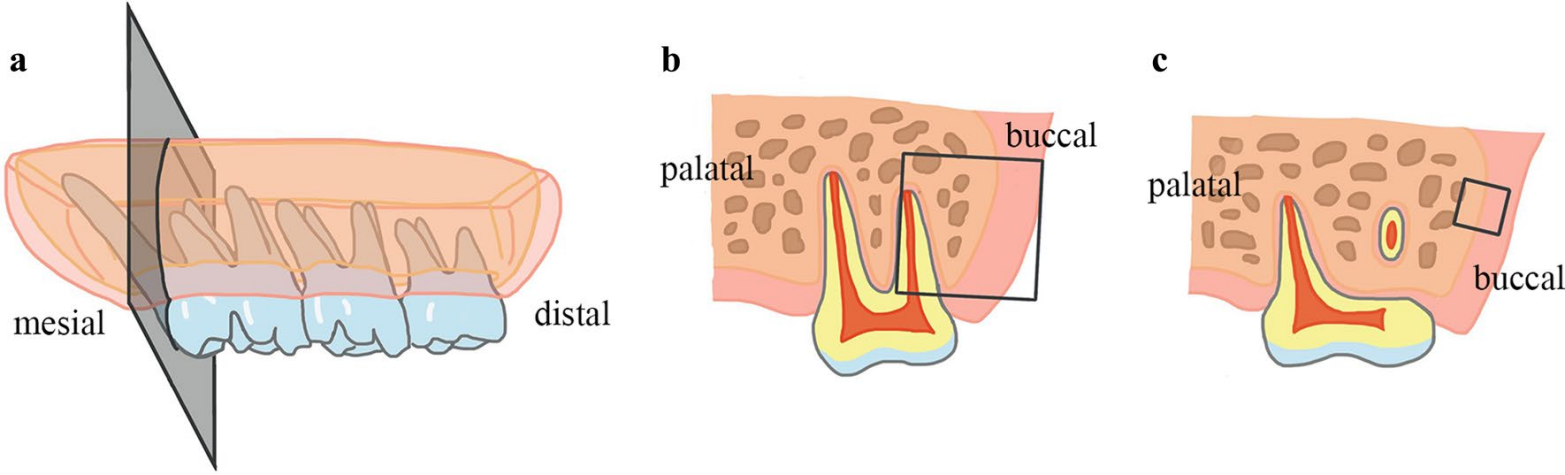
Schematic diagram of undecalcified hard tissue mills and decalcified tissue sections. (Figures were drawn using Tayasui Sketches, version 23.6.). **a** Schematic diagram. **b** Hard tissue mill observation area. **c** Histological section observation area

Five rats from the indicated subgroups were sacrificed by cervical dislocation on the 1st, 3rd, 5th and 7th days after the application of force. Tetracycline hydrochloride (25 mg/kg) was injected into the thigh muscle 24 h before sacrifice. One rat from the control group was randomly selected to be sacrificed without the administration of tetracycline. The maxillary first molar and its surrounding soft and hard tissues were collected. Undecalcified hard tissue mills with thicknesses of approximately 300–500 μm, including the first molars in the proximal buccal root tip area, were made perpendicular to the occlusal surface of the first molars. Five-micrometer-thick decalcified tissue sections were prepared, which were also perpendicular to the occlusal surface of the first molars. Fifteen sections made after the first proximal buccal root appeared were retained; 2 pieces that had intact buccal periosteum were selected for HE staining and 5 pieces for immunohistochemical staining (Fig. 2). The distribution of tetracycline fluorescence in the corresponding periosteum of the root tip area was observed, and HE staining and TGF-β1 immunohistochemical staining were performed.

The immunohistochemical staining procedure was as follows:

A. Paraffin sections were routinely dewaxed and washed with PBS 5 times for 5 min each.
B. The sections were placed in citric acid solution and heated in a water bath at 95 °C for 15 min. When the solution reached room temperature, the sections were washed with PBS 5 times for 5 min each.
C. Endogenous peroxidase blocking agent was added according to the instructions of the secondary antibody kit, incubated for 10 min at room temperature in the dark, and washed with PBS 5 times for 5 min each.
D. Goat serum was added according to the instructions of the secondary antibody kit and incubated at 37 °C for 40 min, and then the serum was removed.
E. TGF-β1 polyclonal antibody (1:400 dilution) was added and incubated overnight at 4 °C. As a control, 10% goat serum was added and incubated overnight at 4 °C.
F. The sections were removed 18 h later, placed at room temperature for 1 h to warm and were washed with PBS 5 times for 5 min each.
G. The reaction enhancement liquid was added according to the instructions of the secondary antibody kit, incubated at 37 °C for 20 min, and washed with PBS 5 times for 5 min each.
H. Enhanced HRP-conjugated goat anti-mouse/rabbit IgG polymer was added according to the instructions of the secondary antibody kit, incubated at room temperature in the dark for 20 min, and washed with PBS 5 times for 5 min each.
I. DAB was added and incubated for 1 to 2 min, and the reaction was terminated with water.
J. Hematoxylin was added and incubated for 10 s, and the reaction was terminated with water.
K. The sections were dehydrated with gradient alcohol, cleared with xylene, and sealed with neutral gum.
L. Microscopic observations showed an intact buccal periosteum, and cells with specific brownish-yellow staining were considered positive.

### Data acquisition

For the immunohistochemical staining sections, Image-Pro Plus software was used to randomly select 3 circular regions within the area that had dense staining in the periosteum adjacent to alveolar bone. The ratio of the integrated optical density (IOD), which represents the intensity of immunohistochemical staining, to the area of interest was measured. The average value was taken as the average integrated optical density of this region.

### Statistical analysis

SPSS 22.0 software was used to conduct multivariate tests and LSD tests for the immunohistochemical staining results of group A, group B and group C at each time point. P < 0.05 was considered statistically significant.

## Results

A total of 60 rats were included in the study. One rat in groups B and C died before the time of sacrifice in the 3-day group, while 1 rat in group B and 2 rats in group C died before the time of sacrifice in the 5-day group; all of these rats were excluded from the statistical analyses. All devices remained in position until the rats were sacrificed.

**Fig. 3:**
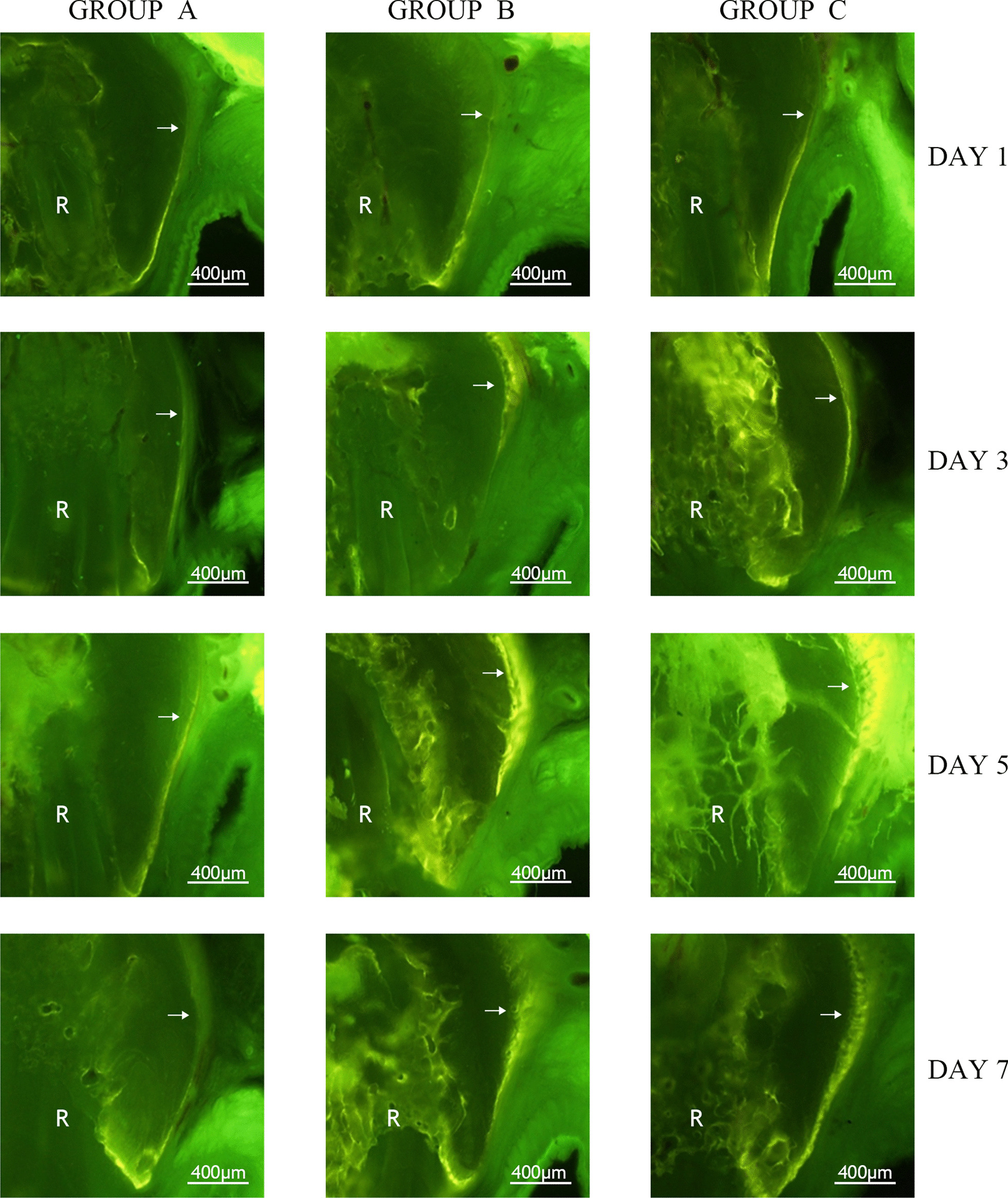
Results of tetracycline fluorescence staining in the first molar buccal apical areas (100 ×). R: Root. The white arrows indicate cortical bone. (Figures were taken using Olympus Cellsens Strandard 2.1 and edited using Adobe Photoshop CS6, version 13.0*64.)

In all the groups, including group A, which was not subjected to experimental intervention, different degrees of tetracycline fluorescence were observed in the alveolar crest area. One day after the application of force, no significant tetracycline fluorescence was observed in the cortical bone corresponding to the buccal root tip areas in rats in group B and group C compared with those in group A. Over time, a strong tetracycline fluorescence signal was observed in the cortical bone corresponding to the buccal root tip areas of rats in group B and group C on the 3rd, 5th and 7th days after the application of force (Fig. 3).

**Fig. 4:**
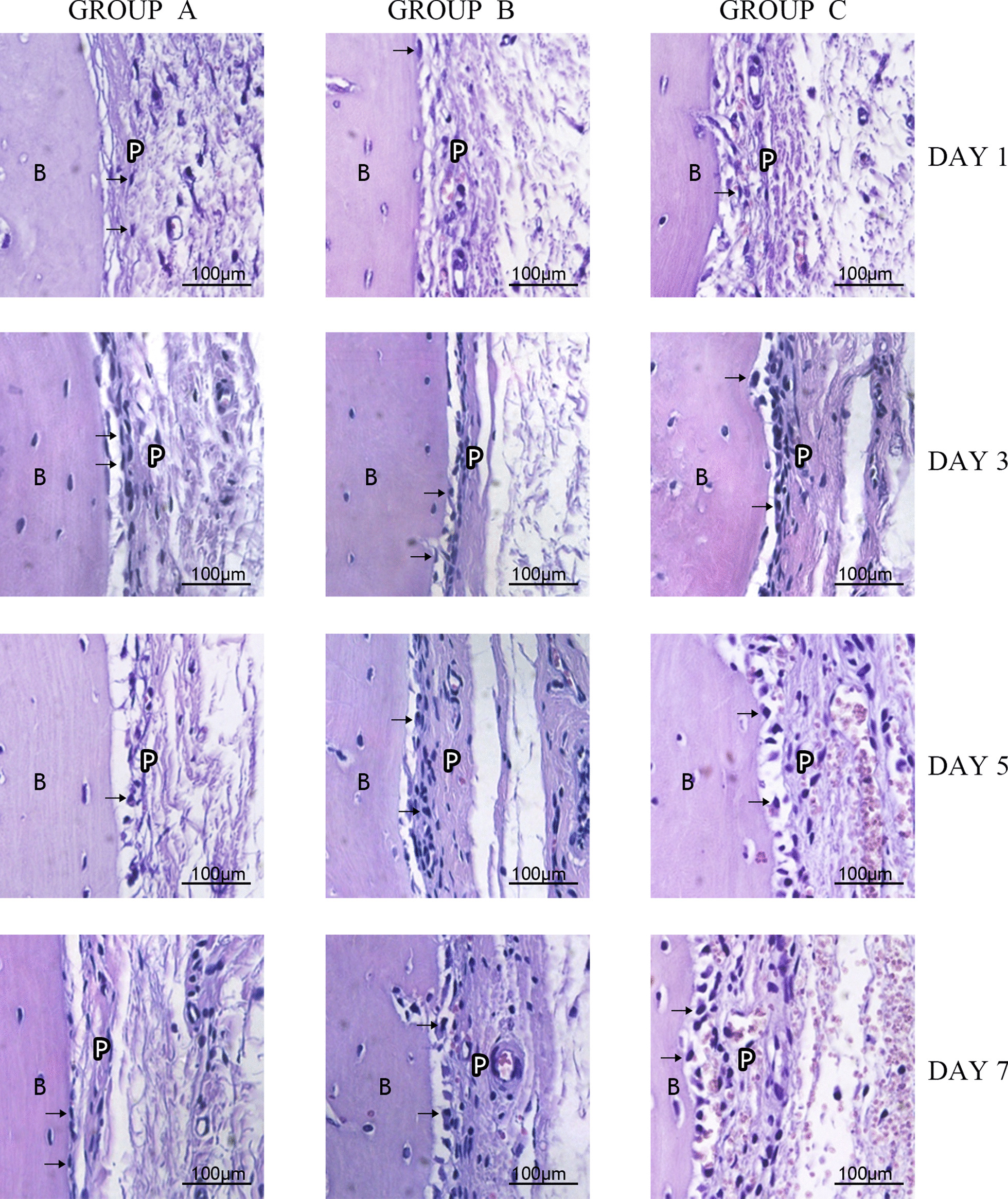
HE Staining Results (400 ×). B: Alveolar Bone. P: Periosteum. The black arrows indicate osteoblasts/preosteoblasts. (Figures were taken using Olympus Cellsens Strandard 2.1 and edited using Adobe Photoshop CS6, version 13.0*64.)

After 1 day of force application, the morphologies of cells (osteoblasts/preosteoblasts) in the periosteum were relatively similar in rats in group A, group B and group C. In the 3-day, 5-day and 7-day groups, cell morphologies were much closer to cube-shaped than those in group A, which were relatively flat (Fig. 4).

**Fig. 5:**
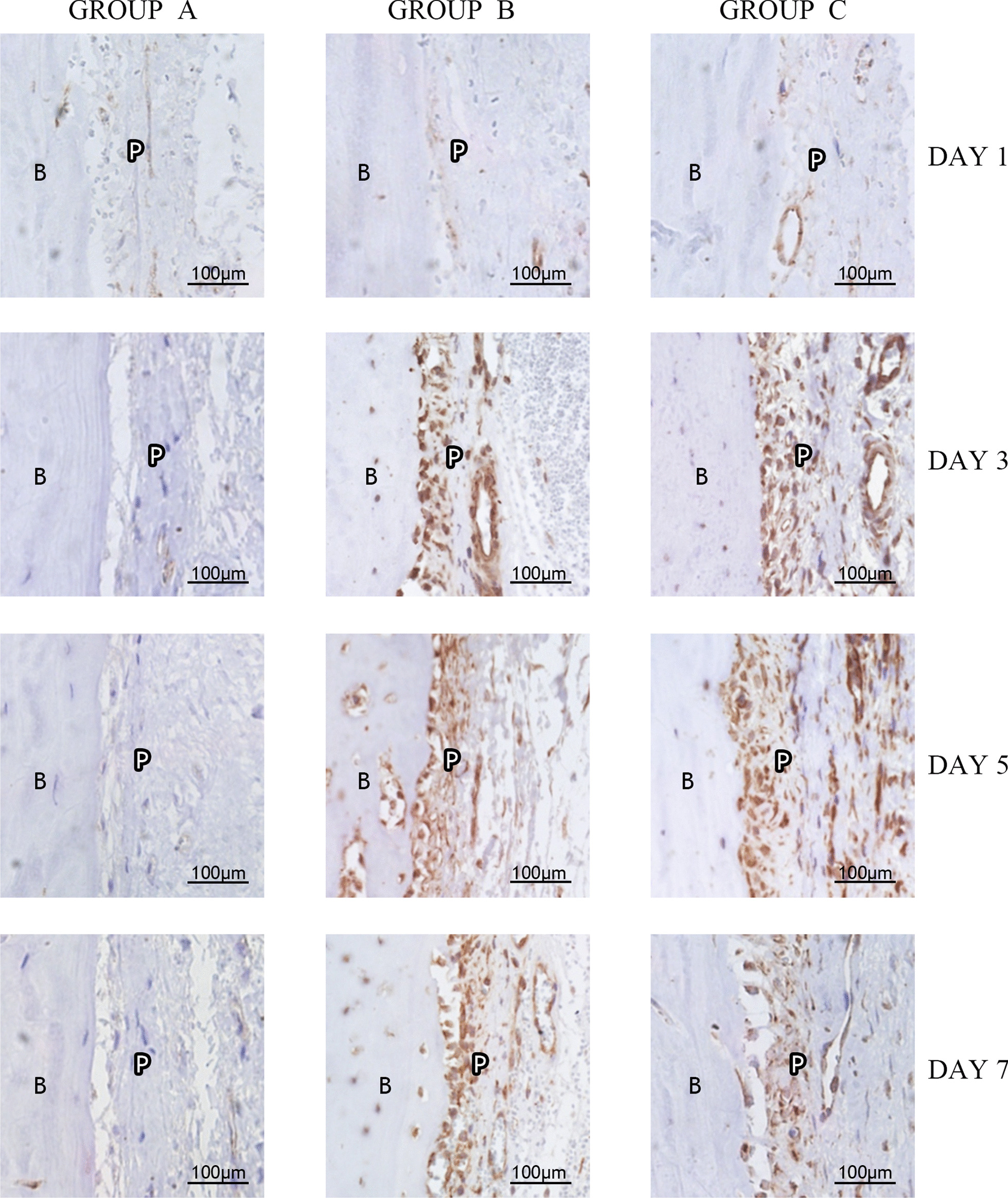
Immunohistochemical staining results of TGF-β1 in the first molar buccal apical region (400 ×). B: Alveolar bone. P: Periosteum. (Pictures were taken using Olympus Cellsens Strandard 2.1 and edited using Adobe Photoshop CS6, version 13.0*64.)

One day later, there was no significant increase in the expression of TGF-β1 in the periosteum in any of the groups. After 3, 5 and 7 days, the expression level of TGF-β1 in the periosteum in group B and group C was significantly increased (Fig. 5). Three sections for each group were chosen for further analysis by Image-Pro Plus (Table 1).

**Table 1:** Average optical density values at different times

	Group(A)	Group(B)	Group(C)
Day1	0.00410 ± 0.00075	0.00465 ± 0.00165	0.00497 ± 0.00104*
Day3	0.00397 ± 0.00036	0.03757 ± 0.00139	0.06638 ± 0.01962*
Day5	0.00415 ± 0.00024	0.07013 ± 0.01938	0.07222 ± 0.00563*
Day7	0.00417 ± 0.00015	0.05136 ± 0.00315	0.05466 ± 0.00594*

(A): Control Group. (B): Orthodontic Group. (C): Corticotomy Group

* Compared to group A and group B, p < 0.05

Multivariate analysis showed that for the effect of time, the p values were lower than the threshold level of 0.05, indicating that time had an effect on the expression level of TGF-β1 in the periosteum. The p values for time and groupings were lower than the threshold level of 0.05, which suggested an interaction between the time and groupings. The LSD test was used to perform pairwise comparisons among the three groups, and the results showed that the p values were all lower than the threshold level of 0.05, suggesting that the pairwise differences among the three groups were statistically significant.

## Discussion

According to the classic theory, when teeth are moved within the alveolar bone by orthodontic force, absorption and accretion of the alveolar bone near the periodontal membrane occur. At the same time, the corresponding surface of the bone also undergoes compensatory remodeling to maintain the same alveolar bone shape and thickness before and after orthodontic intervention [26]. In this study, the histological results showed that this compensatory remodeling did occur during the application of force. Combined with previous studies from this research group and CBCT data from other scholars [1, 27], it can be concluded that compensatory remodeling of the alveolar bone surface occurs, but the rate of remodeling clearly cannot match the rate of tooth movement, which is consistent with previous research results. Edwards suggested that teeth could move within cancellous bone but that the apical tip should not exceed the position of the original palatal cortex [28]. Meikle suggested that when a tooth touches the cortical bone, moving the tooth can cause fenestration and dehiscence [29]. The cortical bone area of the alveolar bone surface is a major factor that restricts orthodontic tooth movement. If we can accelerate compensatory remodeling of the alveolar bone surface and make the rate of bone formation match the rate of tooth movement, this would be of great benefit to orthodontic treatments.

The mechanism by which cortical osteotomy accelerates tooth movement is currently believed to be via the regional acceleratory phenomenon [18], which refers to the rapid acceleration of metabolic activity in local soft and hard tissues when the tissues are subjected to relatively strong stimulation. Some scholars established a mouse model of cortical bone defects in the femur. During the subsequent healing process, it was observed that osteogenesis occurred in the damaged area and that extensive subperiosteal osteogenesis occurred in the adjacent areas [30]. This result suggests that corticotomy can activate bone remodeling in the area near the trauma. This finding is consistent with the results of our study, which showed that corticotomy accelerates the remodeling of the alveolar bone surface under the same orthodontic force.

Remodeling of the cortical bone on the surface of bones is generally considered to be closely related to mechanical forces. In one study of osteoporosis, researchers measured an increase of 25% in cortical bone tissue density after mice were shaken for 15 min a day [31]. Some scholars administered electrical stimulation to a mouse model of congenital osteogenesis imperfectum to make the muscle contract, and the researchers found that the cortical bone tissue density increased significantly [32]. Another study showed that the mandible cortical bone of prehistoric humans with greater chewing load was significantly thicker than that of modern humans and that the lateral lingual cortical bone of the mandible joint, where chewing causes the greatest tensile stress, was significantly thicker [33]. A study of the metacarpal bones of horses found that the cortical bone on the side with more strain was significantly thicker than that on the stressed side and had relatively higher mineral contents [34]. In our study, it was believed that orthodontic force applied to teeth could transform the cortical bone on the surface of alveolar bones, and this hypothesis was consistent with previous research results. Wainwright moved the incisor root tips of monkeys out of cortical bone and made tissue sections. Newly formed cortical bone was found near the fenestration area, but the root tip was not completely covered [35, 36]. Srteiner and Engelking performed global labial movement with an orthodontic device placed on anterior teeth in monkeys and readducted the anterior teeth after the root broke through the cortical bone to cause bone cracking. The results showed that the degree of bone cracking was significantly reduced after the tooth was moved back to the initial position, and the alveolar bone height was partially restored [37, 38].

However, the role of corticotomy in cortical bone regeneration is still controversial in academic circles. When corticotomy is combined with bone graft material, scholars tend to believe that corticotomy enhances the local blood supply, enabling the graft material to be better integrated in the early stage. Danesh-sani studied implant patients who needed bone grafts and found that the number of local microvessels in the corticotomy group was significantly higher than that in the control group when the same graft material was used; in addition, the number of new bones was slightly higher than that in the control group, but this result was not statistically significant [39]. Acar believed that corticotomy had a positive effect on guided bone augmentation in rabbits [40], while Gutta believed that cortical perforations did not have any effect on the quantity of regenerated bone in dogs [41]. Greenstein suggested in a systematic review that it was impossible to determine whether corticotomy improved bone regeneration because there was no uniform standard between studies, and it was entirely up to clinicians to determine effectiveness [42].

Tetracycline is a basic compound in the tetracycline family of antibiotics. It is a broad-spectrum antibiotic. Because tetracycline can be deposited in newly formed bone tissue and teeth, it is used in basic experiments. Under a fluorescence microscope, the areas marked by tetracycline were identified as active osteogenic regions. In this study, tetracycline injection was performed in each group 24 h before the rats were sacrificed, and the fluorescent staining results showed local alveolar bone formation on the corresponding days. The results showed that on the first day, the alveolar crest area in each group, including the control group, showed osteogenic activity, which may be because the alveolar crest itself is a relatively active remodeling area and exhibits osteogenic activity with daily mastication. Three days after treatment, rats in group B and group C showed fluorescence on the external surface of alveolar bone corresponding to the root tip, indicating that relatively active osteogenesis occurred in this area. Therefore, it can be concluded that the presence of orthodontic force causes the surface of alveolar bone to undergo compensatory remodeling.

Consistent with the tetracycline fluorescence staining results, the HE staining results showed that at the cellular level, with the extension of the loading time, the cellular morphology in the periosteum germinal layer on the alveolar bone surface in rats in group B and group C gradually changed from flat to cubic, indicating that under the effect of orthodontic force, osteoblasts began to differentiate. Immunohistochemical staining showed the cytokines involved in this process and quantitatively observed this phenomenon.

Immunohistochemical staining of alveolar bone periosteum in the buccal apical regions of rats showed that TGF-β1 expression in the periosteum-generating area of the apical regions subjected to orthodontic force and corticotomy was significantly increased compared with that in control rats, except on the first day. This finding suggests that TGF-β1 is involved in bone remodeling in the early stages of postconditioning. The peak on the 5th day may be due to further activation of additional latent complexes by previously activated TGF-β1. As the loading time increased, due to the limited spring elongation, the force value may have been attenuated on the seventh day, indicating that the expression of TGF-β1 on the seventh day was decreased compared with that on the fifth day. These results suggest that compensatory remodeling of alveolar bone surfaces occurs and that TGF-β1 participates in this process. Statistical analysis showed that there were differences among group A, group B and group C, and it was not difficult to see that the main difference between group B and group C was that the expression of TGF-β1 peaked earlier in group C than in group B. This result may have occurred due to the presence of trauma; thus, additional osteoblasts and osteoclasts near the wound were recruited to participate in repair, and the local TGF-β1 concentration increased accordingly. The implementation of corticotomy may have resulted in earlier peak expression of TGF-β1 in the osseous surface of alveolar bone corresponding to the buccal apical region than that observed in the control group.

Transforming growth factor-β1 is a multipotent cellular peptide with a molecular weight of 25 kDa, and it is widely present in bone and bone matrix. TGF-β1 can transform mesenchymal cells and contributes to osteocyte growth. TGF-β1 is secreted by a variety of cells, including osteoblasts, fibroblasts, and macrophages, and its receptor is present on many cell surfaces. The TGF-β/Smad signaling pathway is the main pathway through which TGF-β1 mediates its biological effects [43]. In addition, TGF-β can also activate the MAPK signaling pathway and the PI3K/Akt signaling pathway and play an important role in cell proliferation, differentiation, apoptosis and other processes.

Although the results of this study showed significant differences, there were some limitations in evaluating the effect of corticotomy on the compensatory remodeling of alveolar bone. In the future, more experiments should be carried out, including expanding the sample size, extending the observation time and increasing the measurement index to further confirm the current findings.

## Conclusion

Compensatory remodeling of alveolar bone surfaces occurred during the buccal palatal movement of orthodontic teeth, corticotomy had a positive effect on this compensatory remodeling, and TGF-β1 was involved in this process.

## Data Availability

The dataset used and/or analyzed during the current study available from the corresponding author on reasonable request.

## Abbreviations

TGF: Transforming growth factor
CBCT: Cone beam computed tomography
RAP: Regional acceleratory phenomenon
BMP: Bone morphogenetic protein
SPSS: Statistical package for social sciences
LSD: Least significant difference
MAPK: Mitogen-activated protein kinase
